# Sickle Cell Disease: New Opportunities and Challenges in Africa

**DOI:** 10.1155/2013/193252

**Published:** 2013-09-19

**Authors:** J. Makani, S. F. Ofori-Acquah, O. Nnodu, A. Wonkam, K. Ohene-Frempong

**Affiliations:** ^1^Department of Haematology and Blood Transfusion, Muhimbili University of Health and Allied Sciences, P.O. Box 65001, Dar es Salaam, Tanzania; ^2^Nuffield Department of Medicine, University of Oxford, Oxford, UK; ^3^Department of Pediatrics, Emory University School of Medicine, Atlanta, GA, USA; ^4^School of Allied Health Sciences, College of Health Sciences, University of Ghana, Ghana; ^5^Department of Haematology and Blood Transfusion, College of Health Sciences, University of Abuja, Abuja, Nigeria; ^6^Division of Human Genetics, Faculty of Heath Sciences, University of Cape Town, South Africa; ^7^Faculty of Medicine and Biomedical Sciences, University of Yaoundé I, Cameroon; ^8^Children's Hospital of Philadelphia, Philadelphia, PA, USA

## Abstract

Sickle cell disease (SCD) is one of the most common genetic causes of illness and death in the world. This is a review of SCD in Africa, which bears the highest burden of disease. The first section provides an introduction to the molecular basis of SCD and the pathophysiological mechanism of selected clinical events. The second section discusses the epidemiology of the disease (prevalence, morbidity, and mortality), at global level and within Africa. The third section discusses the laboratory diagnosis and management of SCD, emphasizing strategies that been have proven to be effective in areas with limited resources. Throughout the review, specific activities that require evidence to guide healthcare in Africa, as well as strategic areas for further research, will be highlighted.

## 1. Introduction

Sickle cell disease (SCD) consists of a group of disorders characterised by the presence of sickle haemoglobin. Although over 700 structural hemoglobin (Hb) variants have been identified, only two (Hb S, Hb C) reach high frequencies in Africa. The common SCD syndromes in this region include homozygous HbSS disease (HbSS) commonly known as sickle cell anaemia (SCA) and Hb SC disease. SCD was known in some parts of Africa before the twentieth century: inhabitants of western Africa gave the disease-specific names that evoke acute, painful episodes or death or refer to children destined to die and to be reborn as their own siblings [1, 2]. Africa is the major origin of the sickle (*β* ^S^) mutations [3]. There are four chromosomal haplotypes that are associated with the *β* ^S^ mutation. They are named after the regions where they have the highest frequency: Benin, Senegal, Bantu (Central African Region (CAR)), and Arab-Indian. The haplotypes are defined by restriction fragment length polymorphisms (RFLPs) in the *β*-globin locus. Due to the population specificity of the haplotypes, it is believed that the sickle cell mutation arose independently in these populations and remained to this day [4].

### 1.1. Normal Human Hemoglobin

**Figure 1:**
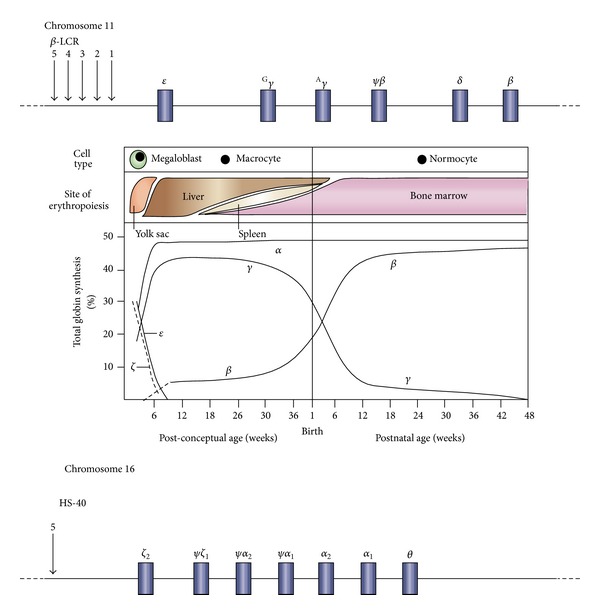
Developmental control of human haemoglobin (Hb) expression [6].

Human Hb is encoded by a cluster of genes located on chromosomes 11 and 16 that are expressed in a developmentally regulated manner. They are tetramers of two pairs of *α*-like and *β*-like globin chains. Adult and fetal hemoglobin have *αβ*(Hb A,  *α*_2_ *β*_2_),  *δ*(Hb A2,  *α*_2_ *δ*_2_), or *γ* chains (Hb F,  *α*_2_ *γ*_2_), whereas in the embryo, *α*-like chains—termed *ζ*_*γ*_ (Hb Portland, *ζ*_2_ *γ*_2_) or *ε* *ζ*_2_ *ε*_2_—and *α* and *ε* chains form Hb Gower 2 (*α*_2_ *ε*_2_) (Figure 1) [5].

Embryonic hemoglobin production is confined to the yolk sac. Thereafter the major site of synthesis is the fetal liver. HbF is the predominant type of hemoglobin in fetal life, but around birth there is a switch from fetal to adult globin gene expression, when HbF is gradually replaced by adult hemoglobin, such that by 6 months of age the major Hb is HbA (*α*_2_ *β*_2_). Residual amounts of HbF, however, continue to be synthesized throughout adult life, and the amounts vary considerably, with the majority of adults having less than 1% HbF.

### 1.2. Pathophysiology of Clinical Events

Sickle haemoglobin (HbS) results from a substitution of one amino acid (Valine) for another amino acid (Glutamic acid) at position six of the *β*-globin polypeptide chain. This substitution is caused by a single-base mutation in codon 6 within the *β*-globin gene on chromosome 11, where the sequence GAG occurs instead of GTG.

Due to the abnormal amino acid in the *β*-globin chain, HbS forms long, insoluble polymers when deoxygenated, and the red blood cells (RBCs) containing HbS become less deformable and form a “sickle” shape. It was previously thought that the clinical consequences were simply due to this abnormal, rigid sickle red blood cell occluding small blood vessels. However, there is increasing evidence that the pathogenesis of the various clinical events, both acute and chronic, results from a series of complex mechanisms which are not limited to the RBC [7]. These relate to concentration of HbS and other haemoglobin variants such as HbF within the cell which reduces its ability to polymerise [8], disturbances in the red cell membrane making the cell less responsive to oxidant stress, and altered membrane lipids resulting in increased rigidity [9–11]. Additionally, adhesion molecules such as integrins (*α*_4_ *β*_1_), (*α*_*v*_ *β*_3_), their receptors (VCAM-1, ICAM-4), selectins interact with endothelial cells, RBC, and a variety of soluble proteins within the plasma, such as thrombospondin (from platelets) and von Willebrand factor from endothelial cells to mediate vasoocclusion within the macro- and microvasculature [12–16]. Finally there is compelling evidence of the role of nitric oxide (NO) in SCD [17]. NO is a potent regulator of basal vasodilator tone. It also inhibits the expression of cellular adhesion molecules [18]. The increase in haemolysis in SCD results in an excess of haemoglobin in the plasma, which exceeds the scavenging capacity of haptoglobin. The result is that there is abnormal “cell-free” haemoglobin, which circulates in plasma, binding to and consuming NO, so causing a reduction in the concentration of NO [19]. This results in vasoconstriction, increased adhesiveness of erythrocytes, leukocytes and endothelial cells, and platelet aggregation.

### 1.3. Clinical Events in SCD

**Figure 2:**
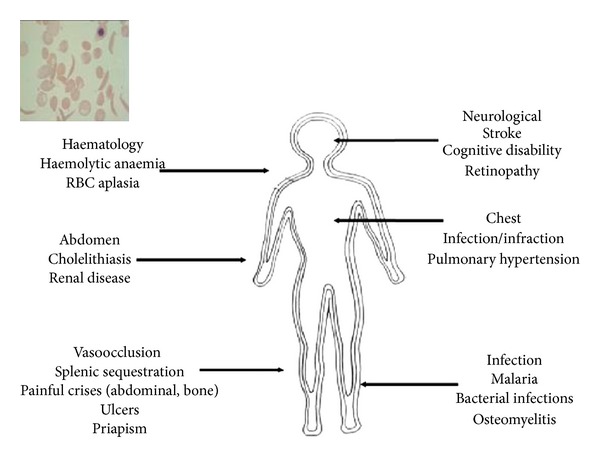
Selected clinical consequences of SCD.

Although SCD stems from an abnormality of the RBC, it is essentially a multisystem disorder, affecting almost every organ system of the body, as shown in Figure 2. The clinical consequences can be divided into 4 groups: haemolysis and haematological complications, vasoocclusion, infection, and organ dysfunction. 

## 2. Haemolysis and Haematological Complications

At birth, individuals with SCD do not have anaemia, but with the synthesis of adult Hb, they develop chronic haemolytic anaemia that is present throughout life. This may be interspersed with acute episodes of reduction in haemoglobin “anaemic crises”. Hyperhemolysis crises are defined by a sudden fall in steady state haemoglobin accompanied by increased reticulocytosis and exaggerated hyperbilirubinemia. The chronic haemolysis in SCD may result in gall bladder disease due to high levels of bilirubin. Although the main cause of anaemia in SCD is chronic haemolysis, there are other types of anaemia that may occur. Acute splenic sequestration, when there is rapid onset of trapping of red blood cells in the spleen, is characterised clinically by a sudden increase in splenic size, at least 2 cm below the left coastal margin, accompanied by a reduction in haemoglobin or haematocrit by 20% of baseline level. This has been described in SCD and is a significant cause of mortality [20]. Anaemia may be secondary to infections such as malaria, bacterial and viral diseases. Of the latter, RBC aplasia in the bone marrow has been notably described and has been associated with infection with parvovirus serotype B19 [21].

## 3. Vasoocclusion

Vasoocclusion (VOC) is thought to be the underlying cause of painful crises, acute splenic sequestration, and priapism (painful and prolonged penile erection). Painful crises, considered the hallmark of SCD, are defined as severe pain lasting for 2 or more hours that is attributable to SCD. The sites that are normally affected include the arms, legs, back, abdomen, chest, and head. Painful crises do not include other causes/types of pain in SCD such as dactylitis, acute chest syndrome, right upper quadrant syndrome, osteomyelitis, and appendicitis. It is the most common cause of hospitalisation and frequent pain (defined as 2 or more painful events a year for three years) is associated with poor quality of life and increased risk of death [22].

## 4. Infection

Individuals with SCD are reported to be susceptible to infections with encapsulated organisms such as *Streptococcus pneumoniae* [23–25]. The use of oral penicillin in the USA had a significant impact on reduction in mortality [26], and it is now policy in many high-income countries to give penicillin prophylaxis and antipneumococcal vaccination to SCD patients [27]. It was previously thought that the situation in Africa may be different. Aside from the fact that the data regarding the clinical spectrum of SCD are limited, there was controversy regarding the role and significance of pneumococcal disease in causing morbidity and mortality in SCD in this setting [28]. However, there is emerging evidence to confirm that pneumococcal disease is a significant cause of bacteraemia in SCD [29], with calls to introduce interventions for preventing infections as a critical factor in improving survival [30, 31]. The various factors that are associated with increased infections in SCD may be directly related or unrelated to the immune system. Some infections may be the result of a complication or treatment of SCD itself. SCD patients are at high risk of transfusion-transmissible infections particularly with human immunodeficiency virus and viral hepatitis since they receive frequent, often unplanned emergency blood transfusion (BT) [32–35]. This is particularly important in Africa, given the high prevalence of HIV infection and the operational problems in providing adequate blood-transfusion services. Long-term BT may result in iron overload, which in itself is associated with infections due to *Yersinia Enterocolitica *[36]. SCD causes end-organ damage to the lung, liver, kidney, and skin, making these sites susceptible to infection by unusual organisms. In addition, skeletal complications, poor perfusion, and blood supply to bone tissue are also thought to contribute to increased susceptibility to infections of the bone, osteomyelitis, which is often due to salmonella infections [37]. Other factors include high bone marrow turnover due to chronic haemolysis which results in increased susceptibility to viral infection. Parvovirus B19 infections are one of the viral infections that predispose to poor outcome with erythrocytic aplasia that may lead to life-threatening anaemia [21, 38, 39]. However, the epidemiology of this virus in Africa is poorly defined [40–42]. Individuals with SCD may have impairment of the immune system, involving both cellular immunity and humoral immunity. The most well-described immune defect is caused by reduced function of the spleen. Patients with SCD have repeated splenic infarction due to vasoocclusion which causes loss of the splenic vasculature leading to hyposplenism [43]. Reports have suggested that 14% patients with SS-SCD are functionally asplenic at 6 months of age, with this number gradually increasing: 28% at 1 year, 58% at 2 years, 78% at 3 years, and by 5 years, 94% are affected [44]. This is from an area without malaria. One of the roles of the spleen is filtration of unopsonised bacteria and remnants of red blood cells from intravascular space as well as opsonised bacteria [45]. Furthermore, the spleen is involved in the synthesis of soluble mediators of immunity. Therefore patients with SCD, with a functional asplenia, have been reported to have impaired antibody responses as well as lacking specific antibodies, particularly against *Salmonella *species and *Streptococcus pneumonia* [46]. This is thought to be due to deficiency of a complement factor involved in the activation of the immune system. The classic pathway is activated by antigen-antibody interaction which causes fixation of complement components C1, C2, and C4 which then activate C3, whereas in the alternate pathway the antigen directly activates C3. Activation of C3, which is an opsonin, results in fixing of antigens on the microorganism [47] making them susceptible to enhanced phagocytosis by neutrophils and monocytes/macrophage. Johnston et al. illustrated that patients with SCD have an abnormality in the activation of this pathway with failure of full activation and fixing of C3 to encapsulated bacteria [48]. This results in failure of SCD patients to phagocytose invading organisms, particularly *Streptococcus pneumoniae.* The distinction between factors directly related to the immune system or not is somewhat arbitrary as there is a lot of overlap between the various factors. Although there have been reports of different patterns of infections in patients with SCD, summarised in Table 1, this review focuses on invasive bacterial infections as detected by blood culture. In the absence of prophylaxis, infections are thought to be the leading cause precipitating clinical events and associated with increased mortality [23, 49].

## 5. End-Organ Dysfunction

With increase in survival, major organs in individuals with SCD are eventually damaged. The brain and lungs are particularly affected, with stroke, defined as an acute neurological syndrome due to vascular occlusion or haemorrhage in which symptoms and signs last for more than 24 hours, being a well-described event. Acute chest syndrome (ACS) is an acute respiratory illness characterised by new pulmonary infiltrates on chest X-ray and falling arterial oxygen saturation [54, 55]. Both these events have been reported to occur with high prevalence in SCD and are also risk factors for death [23, 55, 56]. 

## 6. Heterogeneity of Clinical Events in SCD

The clinical expression of SCD is heterogeneous (Table 2). There is interindividual variability ranging from near complete asymptomatic to severe debilitating illness. There is also variability within an individual, with changes in the type and frequency of clinical events with age. Finally, there is variability in clinical events depending on the geographical location. This is due to the differences in environmental factors such as nutrition, socioeconomic status, and climate that will influence the natural history of disease. The general pattern of clinical disease is characterised by quiescent periods interspersed with acute events, which are referred to as crises.

The reasons for this heterogeneity are not fully understood [94]. Interindividual variation in fetal hemoglobin (HbF) levels is one of the main modifiers that contribute to the clinical heterogeneity observed in SCD patients. Higher expression of HbF in adulthood ameliorates morbidity and mortality in SCD [56, 95].

It is now clear that common HbF variation is a quantitative genetic trait shaped by common polymorphisms. Multiple genes, together with an environmental component, determine the measured value of HbF in any given individual. Genetic variation at three major loci accounts for a relatively large proportion (20%–50%) of the phenotypic variation in HbF levels: (1) a single-base substitution (T/C) at position −158 of the ^*G*^ *γ* globin gene, termed *Xmn*I ^*G*^ *γ* site [96]; (2) the *HMIP* locus (*HBS1L-MYB intergenic polymorphism*) on chromosome 6q [97]; and (3) the oncogene *BCL11A* on chromosome 2 [98]. These variants have been well reported in nonanemic Northern Europeans and Sardinians, a *β*-thalassemia cohort, in SCD patients from Brazil, and in the African-American Cooperative Study of Sickle Cell Disease (CSSCD) [99–101]. There is very little description of the three main genetic polymorphisms explaining phenotypic variation in HbF levels and clinical phenotype in native African SCD patients [97, 102].

## 7. Epidemiology of Sickle Cell Disease

### 7.1. Prevalence

The prevalence of SCD can be objectively determined by calculating the birth prevalence of affected children, which requires accurate diagnosis and registration at birth. Since this is not done in most African countries, an alternative method is to use the prevalence of the carrier or heterozygous states (HbAS) to calculate the expected birth rate of SCA based on the gene frequency and Hardy-Weinberg equation. Approximately 300,000 children are born every year with SCD in the world, and countries such as the United States of America, United Kingdom, and Jamaica have well-documented SCD population. However, this SCD population constitutes only 1% of the global population of SCD, as over 75% are in Sub-Saharan Africa [103, 104]. It has been estimated that SCD results in the annual loss of several millions of disability-adjusted life years, particularly in the developing world [105]. Hemoglobinopathies alone represent a health burden comparable to that of communicable and other major diseases [106].

### 7.2. Population Genetics and Dynamics: SCD, Malaria, and Migration

Compared to noncarriers, healthy carriers of recessive genes for SCD have a well-documented survival advantage against the lethal effects of malaria. As a result, carriers are more likely to reach reproductive age. Consequently, the birth prevalence of SCD is high in Africa [107–109]. The resurgence of malaria in many parts of the world will serve to maintain these polymorphisms, but even if this selective force were removed it would take many generations for the gene frequencies of these conditions to fall significantly [110]. Any changes resulting from variation in selection or population dynamics will, however, be very small compared with the effect of the demographic transition that many countries have undergone over recent years [110]. Specifically, there is a high prevalence of hemoglobin S (HbS) in Africa and hemoglobin C (HbC) in parts of West Africa [111]. Since subjects that are homozygous for HbC do not present with severe disease like HbSS, it is anticipated that the frequency of HbC will progressively increase even if malaria is not controlled [112]. Internal migration in Africa has led to SCD, which was previously rare, being introduced in South Africa through an influx of migrants from West and Central Africa [113]. The high birth prevalence of SCD has highlighted the burden of SCD, such that in 2006, the World Health Organization (WHO) recognized SCD as a public health priority [114]. There is limited information about the burden of SCD to the health system and the impact that it has on individuals.

### 7.3. Mortality

There is a higher rate of mortality among individuals with SCD, with reports suggesting that if untreated most children with SCD die in early childhood. Studies done in Nigeria, reported mortality of up to 90% [115] but recent estimates suggest that mortality rate has decreased and is more likely to be up to 50% by 20 years. This mortality rate in Africa is similar to those reported in the early 1960s in the United States of America and United Kingdom. However, with early diagnosis and comprehensive treatment, significant reductions in mortality have been achieved, with recent reports of improved survival; 85.6% survive to 18 years in the USA [116], 84% in Jamaica, and 99.0% to 16 years in the UK [117]. The common causes of death in the USA, UK, and Jamaica are infections, acute splenic sequestration, and acute chest syndrome [23, 49, 118, 119] with the highest incidence between 1 and 3 years of age.

## 8. Laboratory Diagnosis of Sickle Cell Disease

The laboratory diagnosis of SCD is based on the demonstration of HbS and the absence or significant reductions in HbA, with variation in the percentage of two other hemoglobins—HbF, HbA_2_—in RBCs. Commonly available screening tests in Africa include sodium metabisulphite sickling test and sickle solubility tests and confirmatory tests using electrophoresis and chromatography to confirm the sickle phenotype (SS/AS/SC/S*β* ^−^thalassaemia). The three tests widely used are haemoglobin electrophoresis, isoelectric focusing (IEF), and high performance liquid chromatography (HPLC). DNA-based assays precisely describe the genotype; however, for clinical purposes, diagnosis usually involves screening (sickling or solubility test) followed by confirmation of the sickle phenotype using gel electrophoresis, IEF or HPLC.

### 8.1. Screening Tests

In most African hospitals, screening is done, using the “sickling test”, which involves making a thin blood film which is then put under hypoxic conditions by the addition of sodium metabisulphite. This will result in RBCs containing HbS becoming deformed (i.e., forming sickle cells) as detected by light microscopy. A “positive” sickling test identifies the presence of sickled RBCs, which occurs in both homo- (SS) and heterozygous (AS) states. The sickle solubility test is another method used for screening which is based on the principle that HbS becomes insoluble when it is deoxygenated. Additional confirmatory tests are required to confirm SS-SCD or SCD involving other Hb types, when these screening assays are used.

### 8.2. Confirmatory Tests

These tests are based on the principle that different haemoglobin isoforms have different overall ionic charge, which makes them migrate with different velocities in an electric field. HBE can be done under alkaline or acidic conditions. HbA, HA_2_, HbF, and HbS migrate towards the anode under an electric field with different rate of mobility. During alkaline Hb electrophoresis the resolution between HbS and HbF can be poor, particularly in individuals with high HbF levels, for example, neonates. Under acidic conditions, HbF migrates relatively more rapidly and is therefore distinguishable from both HbA and HbS. Isoelectric focusing uses the same principles but is slightly more expensive than HBE. However, it is able to identify more Hb variants that would not be detected by HBE. It also has the advantage that it does not require commercial reagents. HPLC uses cation exchange chromatography to identify the various hemoglobins in an individual. It has the advantage in that it can also accurately quantify the Hb levels. In resource-rich countries, screening has largely been replaced by HPLC and confirmation is then done by IEF or HBE. This is mainly because HBE and IEF are labour intensive, time consuming and would not identify abnormal bands or quantify Hb. Furthermore, the quantification of Hb fractions by HPLC is used to monitor patients who are on Hydroxyurea therapy or exchange blood transfusion.

### 8.3. Molecular Diagnosis of SCA

**Figure 3:**
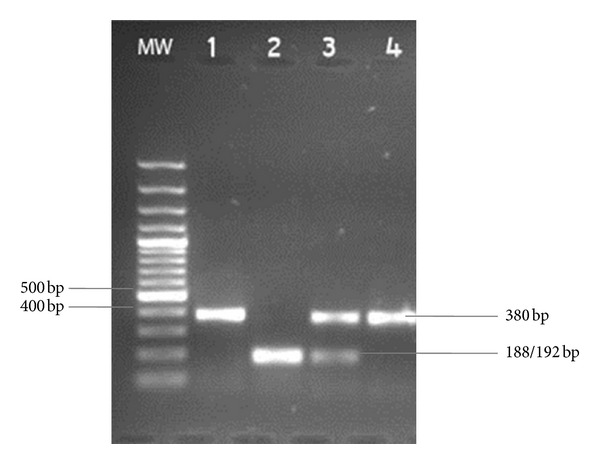
RFLP of HBB fragment with DdeI. Lane 1: undigested control, Lane 2: HbAA control, Lane 3: HbAS control, and Lane 4: HbSS MW: molecular weight marker.

The most popular molecular diagnosis of *β* ^S^ mutation, based on restriction enzyme digestion, is performed on HBB PCR products. The point mutation, which results in SCD, abolishes the restriction site for the restriction enzyme *DdeI*. Digestion of DNA of individuals homozygous for HbAA would result in two fragments 188 bp and 192 bp. Analysis of heterozygous HbAS samples would result in three fragments one of 380 bp and the two digested fragments of 180 bp and 192 bp. Homozygous HbSS samples would result in 380 bp fragments being produced (Figure 3). This method is simple and cost effective and could be used for prenatal genetic diagnosis in African settings [120].

## 9. Management of Sickle Cell Disease

As a chronic disease, the natural history of SCD is characterised by quiescent periods interspersed by acute events, known as crises, leading to patients seeking health care and frequent hospitalisation. The “crises” range from defined syndromes such as acute chest syndrome (ACS), acute splenic sequestration (ASS), to less well-defined symptoms that include pain, fever, anaemia, worsening of jaundice, and leg ulcers. Other circumstances include pregnancy, dehydration, and extreme cold weather. With the increased life span of individuals with SCD, there has been an increasing awareness of the importance of improving the quality of life as well as preventing damage to major organs. SCD is associated with increased mortality. The causes of mortality in the USA, UK, and Jamaica included infections, ACS, ASS, and aplastic crises [23, 49, 118, 119]. The management of patients with SCD involves interventions that improve survival, prevent complications, treat acute events, and reduce end-organ damage. Specific conditions or circumstances when SCD patients require extra care include surgery requiring general anaesthesia, due to increased risk of developing acute sickling complications and sudden death. Over the past 3 decades there has been an improvement in the understanding of the different pathogenic mechanisms responsible for sickle cell events and organ dysfunction. Through a series of clinical trials, effective interventional strategies have been established.

### 9.1. Newborn Screening (NBS)

The highest incidence of death occurs in the first 3 years of life [23, 49, 118, 121]. Identification of children at birth by newborn screening (NBS), and institution of preventative care has improved survival [116, 122, 123]. Patients who are identified at birth can be given counselling and advice about the course of illness. They can then be enrolled in comprehensive care programmes that provide prompt and effective care of acute events and prophylaxis against complications, resulting in overall positive impact on survival and quality of life. Countries with large SCD populations and adequate resources have started NBS programmes.

### 9.2. Comprehensive Care Including Dedicated Day Care Facilities

The identification of SCD at birth has to be accompanied by enrolment into programmes that provide comprehensive care by multidisciplinary teams comprising nurses, genetic counsellors, social workers, paediatricians, haematologists, orthopaedic surgeons, ophthalmologists, and internists. These programmes provide appropriate advice, counseling, and support to parents and affected individuals. This includes advice such as drinking adequate quantities of fluid to avoid dehydration and wearing warm clothing in cold weather. Specific health education that will enable them to recognise acute events and seek medical care is also essential. Teaching mothers to recognise enlargement of the spleen and anaemia was effective in diagnosing and treating anaemia due to ASS [71, 124]. Patients are also seen on a regular basis and provided with folic acid supplements. The evidence for the burden of folate deficiency in SCD is limited. Prompt treatment of crises (fever and pain), particularly at outpatient or in day-care facilities, has been found to be effective and reduces the burden of hospitalization to the individual and the health system [125–128]. Long-term care should be provided by a multidisciplinary team including professionals who have specialized in haematology and blood transfusion for adults and paediatric haematologists in children. In settings where there is a low prevalence of SCD or limited number of health care professionals, SCD patients can receive care from general health care workers. In such a setting, guidelines for management can be provided to general health care workers with a system of referral to specialised centres.

### 9.3. Prevention and Treatment of Infections

In the absence of intervention, bacterial infection is the leading cause of mortality in individuals with SCD, and the age group that is most affected is 1 to 3 years [37, 49, 118]. Bacterial infection in SCD is mainly due to *Streptococcus pneumoniae,* resulting in pneumonia, sepsis, and meningitis. The highest incidence of invasive pneumococcal disease is in children less than 6 years of age [91, 118]. In a landmark study in the USA, Gaston and colleagues demonstrated an 84% reduction in incidence of pneumococcal infection with the use of oral penicillin [26]. Interventions with daily oral penicillin and vaccination against pneumococcal infections have successfully reduced mortality in developed countries [26, 116, 129]. In Africa, these interventions have not been implemented as the evidence to demonstrate a similar role of bacterial infections was lacking. This made it difficult for hospitals and governments in developing countries to implement these interventions. Furthermore, published reports have actually questioned the role of prophylaxis against *Streptococcus pneumoniae* (SPN), in Africa [28]. However, there has been increasing evidence of the role of bacterial infections, particularly due to SPN in causing high childhood mortality [130, 131]. Since SCD patients are highly susceptible to SPN infections due to impaired immunity, this makes it even more likely that SPN infections will have a more significant role in SCD mortality. Therefore, there has been an increase in the appeal to implement these interventions [30, 132].

Malaria is widely considered to be one of the major causes of illness and death in patients living with SCD in SSA [90, 104]. Although, SCD individuals have an element of protection against malaria; with a lower prevalence of malaria infection [133–135] and a lower parasite density [136], the risk of mortality when SCD patients get malaria is significantly higher [137]. It is recommended that individuals with SCD who live in a malaria endemic area should receive prophylaxis against malaria [138]. There is ongoing debate as to what is the most appropriate agent that can be used for chemoprophylaxis. The increasing resistance by *Plasmodium falciparum* parasites to chloroquine has meant that most countries have had to stop using chloroquine. Sulphadoxinepyrimethamine has antifolate properties and is not recommended for prophylaxis in patients with SCD who are considered to be folate deficient. Most malaria-endemic countries have therefore been unable to decide which drug to use for prophylaxis in SCD, with options limited to proguanil (paludrine), mefloquine (Lariam), Malarone, or Doxycycline. Current practice in malaria-endemic countries involves use of insecticide-treated nets and prompt diagnosis and treatment of malaria. 

### 9.4. Blood Transfusion (BT)

SCD is contributing to the anaemia in under fives and pregnant women in areas of high prevalence. Patients with SCD have a compensated chronic haemolytic anaemia which allows them to carry on with normal activities at steady-state haemoglobin with narrow reserve capacity to accommodate strenuous physical activities. The steady state haemoglobin varies from person to person and is related to the level of HbF, co-inheritance of alpha thalassaemia, or heterozygosity for another haemoglobin type such as HbC. Although individuals with SCD have chronic anaemia which is tolerated, rapidly worsening anaemia can occur, and this presents as an emergency. It can be caused by ASS, aplastic crises, and hyperhemolysis or associated with other events such as bacterial infections and malaria. Under these circumstances, anaemia is life threatening and requires prompt treatment with blood transfusion. The products that are used (whole blood or packed RBCs) and the method of transfusion (simple or exchange) are determined by the clinical situation, availability of resources, and the capacity to provide the blood product and establish venous access [139]. Blood transfusion is also effective in other situations, such as acute stroke [140], ACS [141], and perioperatively [142]. Blood transfusion works by increasing the level of Hb, thus improving oxygen delivery. It also reduces the proportion of sickle RBCs in the circulation. Exchange or red cell transfusion has also been shown to be effective in reducing the level of HbS to less than 30% [143–146]. This is thought to reduce the deleterious effects of HbS and improve outcome. Long-term blood transfusion therapy (LTBT) has been found to be effective in the prevention of brain injury due to cerebrovascular disease [140]. Blood transfusion is associated with risks which have to be weighed against the benefits when considering implementing this as an intervention. These will be reviewed in the section on stroke.

### 9.5. Pain

Pain, the defining feature of SCD and its commonest symptom, starts early in life and persists throughout life. It is the commonest symptom of SCD and is related to disease severity. Studies in children in developed countries suggest that painful episodes and acute chest syndrome were the most frequent complications of sickle cell disease and that the pain crises are a major predictor of adverse outcome in children along with anaemia and leucocytosis. In adults, large proportion of patients die during an acute episode of pain, making it a risk factor for early death along with acute chest syndrome and stroke. However due to its subjective nature, patients with SCD may not be having appropriate assessment and adequate pain management necessary to prevent complications relating to the pain such as the development of a chronic pain syndrome resulting in worsening of the sickle cell condition. Training is essential for adequate assessment of pain intensity, reporting, documentation by patients, care giver, and health workers. Prompt management of pain requires attention to the precipitating causes (stress, infection, dehydration, acidosis, and allodynia). Adequate oral analgesic should be administered for mild pain and parenteral for moderate to severe pain according to WHO step ladder for analgesia in patients. When the expected relief is not obtained in response to adequate doses of analgesics, this should alert to the condition of opioid-induced hyperaesthesia, allodynia, or the progression of acute pain to chronic pain [147–149]. However, many health facilities in Africa do not have access to opioids.

### 9.6. Hydroxyurea

Hydroxyurea (HU) (also known as hydroxycarbamide) has been reported to be effective in improving survival and reducing morbidity in some SCD patients (Table 3). The clinical outcomes include reduction in frequency of painful episodes and hospital admissions [150]. Hydroxyurea therapy is also monitored by a number of laboratory parameters which include increased HbF levels, mean corpuscular volume (MCV), and reduction in WBC count. Hydroxyurea has been found to be effective in the prevention of brain injury due to cerebrovascular disease [151].

### 9.7. Nitric Oxide

Lung dysfunction results from a combination of repeated pulmonary infections and infarctions as well as increased vasoconstriction leading to pulmonary hypertension [54, 55]. The latter has recently been shown to be associated with reduced bioavailability of nitric oxide [19], which has resulted in the development of potential therapies such as L-arginine, citrulline, and inhaled nitric oxide which is aimed at increasing NO levels through different pathways [153–157].

### 9.8. Stem Cell Transplant

The only cure that is available for SCD is stem cell transplantation (SCT), which replaces the host's bone marrow with stem cells containing normal *β*-globin genotype. Since the first successful transplant reported in 1984 [158], there has been significant reduction in risks due to SCT and increasing success, with the best results, of up to 85% event free survival, occurring with HLA-matched sibling donors and transplantation early in the course of the disease before end-organ damage occurs [159]. One limitation of SCT is the availability of sibling donors [160], and therefore there have been attempts to improve survival for unrelated stem-cell donors [161, 162]. The second limitation of SCT is that this line of treatment requires tremendous resources, and it becomes increasingly difficult for transplant physicians practicing in the developing world to reconcile the difference between what is possible and what is available. Moreover, it is more difficult to address because the clinical course of SCD is extremely heterogeneous. Despite the knowledge of various genetic and environmental factors known to alter disease severity, it is still difficult to accurately identify children with risk of severe disease before extensive damage has occurred. Until such time that a low-risk, definitive cure is available, the cornerstone of management of SCD is the prevention of early mortality, prevention of end organ damage, and improvement of the quality of life.

### 9.9. Gene Therapy

Since SCD is caused by a defective gene, definitive treatment would involve replacement of this gene with a normal gene. This has been done successfully in the sickle transgenic mouse [163], but progress in humans has been limited by identification of appropriate vectors and efficacy for gene transfer and low level expression of globin genes.

### 9.10. Role of Programmes for Control and Management of SCD

From a public health perspective, the policy for approaching the control of SCD in national health programmes needs to work in the context of countries with limited resources in health. Although, there is ongoing debate whether care of SCD should be integrated into existing health care services or whether there should be separate disease-specific programmes for SCD, the WHO recommends [164] that, for countries where the birth rate of affected infants is above 0.5 per 1,000 births, they should develop separate programmes for these conditions. It is recommended that counties with a high prevalence of SCD start planning effective control measures. In this context, control of SCD encompasses two elements: providing best possible care for affected individuals and preventing the birth of affected individuals.

With regard to providing best possible care, the following are options, depending on available resources, that have been recommended by Weatherall et al. in 2006 [105].

 
*Option one*: best possible patient care with the use of prophylactic penicillin following diagnosis, together with retrospective genetic counselling.
 
*Option two*: best possible patient care, together with a newborn screening program and the use of penicillin for all homozygous babies, together with retrospective screening and counselling.
 
*Option three*: best possible patient care, together with newborn screening and the use of prophylactic penicillin from birth for homozygotes, together with population screening and prospective genetic counselling.
 
*Option four*: option three, plus the availability of prenatal diagnosis, bone marrow transplantation, or both.

The management of SCD involves early diagnosis of affected people, the provision of the most appropriate basic, cost-effective treatment, and genetic counselling and psychosocial support. The long-term goal is to ensure appropriate management at different levels of health care with development of referral centres for specialised diagnosis and treatment. This approach ensures a cost-effective way of effectively dealing with a highly prevalent condition in areas where the resources are limited. However, it is important that these centres are not limited to urban areas or centred on academic or research oriented health facilities. In order to avoid this, there must be active strategies to ensure that appropriate management is built into services at all levels of health care with adequate support from these specialised centres. Management of SCD needs to be accompanied by strategies that aimed at two levels of prevention: tertiary prevention which involves early diagnosis of SCD and prevention of complications and more ambitiously secondary prevention which tries to reduce the number of children that are born with SCD. (Note that primary prevention aims to ensure that individuals are born free of SCD). Preventative services involve community education, population screening, and genetic counselling that would encourage people to undergo screening before conception, during the antenatal or postnatal period. There are several issues that need to be addressed with regard to prevention of SCD. The aim of screening is to detect SCD in the foetus, discuss the consequences of a diagnosis of SCD, and provide options for treatment and prognosis. Since SCD is a recessive disorder, during pre-conception screening, the chances of getting an affected child are variable. There is difficulty in advising a couple not to have children as the risk of getting an affected child may be relatively low (1 in 4) and does not increase with each pregnancy. The highest risk would be for two individuals who are SS who wish to have children. This is different from thalassaemia, where children with the most severe form, thalassaemia major, will inevitably have severe disease. Therefore, one could argue that this therefore justifies the use of prenatal diagnosis as this would identify pregnancies with SCD children, and then parents would be given the appropriate information regarding the consequences and prognosis of SCD and allow more reproductive options to families. Prenatal genetic diagnosis represents one type of reproductive option as it provides parents with the option to test at-risk pregnancies and make decisions regarding affected pregnancies. The availability and acceptability of prenatal diagnosis and termination of an affected pregnancy are of particular importance in low-resource countries where neither health services nor families can afford to pay for long-term treatment of SCA [165]. Close to two-thirds of a sample of 130 Cameroonian parents with affected children reported they would accept termination of an affected pregnancy for SCA [120], a considerably higher proportion when compared to the Cameroonian preclinical, clinical medical student, and physicians in a previous study (22.4, 10.8 and 36.1%, resp.) [166]. Trends reported in Nigerian parents were slightly different where 92% of a sample of 53 SCA heterozygous carrier mothers favored prenatal diagnosis and 63% indicated they would opt for termination of an affected pregnancy [167]. However, in a survey of 403 health workers in a tertiary health care centre in Nigeria, only one-third of the respondents accept termination of pregnancy as an option if prenatal screening is positive for SCA, whereas close to half of the respondents (42%) were against the idea. Another study reported that 21.4% of Nigerian doctors would accept termination of an affected pregnancy for SCA [168]. Experience of the effective practice of prenatal genetic diagnosis for SCD (amniocentesis and fetal DNA analysis) was reported in Nigeria and Cameroon [169, 170]. The views of parents towards prenatal diagnosis and in some cases medical termination of pregnancy may be associated with their experience of affected patients and the psychosocial and/or economic impact of SCA on families. Nevertheless the discrepancy between perception of a professional and parents underscores the necessity for more studies to unravel the ethical dilemma around prenatal genetic diagnosis to offer a service that does not conflict with social and cultural values of the affected population. Preimplantation genetic diagnosis is a mechanism for accurate genetic diagnosis, careful selection of unaffected embryo and implantation to allow fertile or infertile couples to have offspring without SCA. It is an expensive procedure using assisted conception by in vitro fertilization or intracytoplasmic sperm injection. It requires close collaboration between fertility specialists, molecular biologists, geneticists, and genetic and fertility counselors and may be an option to individuals who may object to prenatal diagnosis followed by termination.

Although SCA is the most severe form of the disease (compared to SC/S*β* thalassaemia, etc.), there is still wide variability in disease severity. Therefore, even with the correct identification and diagnosis of SS with screening, it would be difficult to predict those who would develop severe disease and have a poor outcome.

## 10. Conclusion and Future Challenges

Because of their uneven distribution in high-frequency populations, reflecting their complex population genetics, the true magnitude of burden of SCD is still unknown. In many African countries there are few or virtually no facilities for appropriate diagnosis and management of SCD. There is limited data about frequency, clinical course, or mortality. Without this information it will be impossible to persuade African governments about the burden of this disease. The WHO Africa has recommended a set of public health interventions to reduce the burden of SCD in African region, namely, improving awareness, preventing the disease, early detection, improving the provision of health care for affected individuals by providing effective clinical, laboratory, diagnostic, and imaging facilities adapted to different levels of the health system, screening of newborns, training of health care workers, developing protocols for treatment, providing genetic counseling, patient support groups, advocacy, and research [171]. The situation will be improved by commitment by member states to integrate SCD prevention and control in national health plans and provide conducive environment for various stakeholders to contribute to the reduction of SCD prevalence, morbidity, and mortality. It will also require concerted action on the part of the international community of the richer countries, together with input from other major international health organizations and funding agencies [172, 173].

## Figures and Tables

**Table 1:** Clinical syndromes and common causative organisms reported in SCD.

Syndrome	Organisms	Reference
Septicaemia	*S. pneumoniae, H. influenza, Salmonella* spp, *E. Coli, S. Aureus*, and *M. Pneumoniae *	[28, 29, 50]
Pneumonia	*S. pneumoniae*, *M. Pneumoniae*, *Chlamydiae pneumonia *	
Meningitis	*S. pneumoniae *	
Osteomyelitis	*Salmonella* spp., *E. Coli*, Gram negative organisms, and *S. Aureus *	[37, 51, 52]
Aplastic anaemia	Parvovirus	[21, 38, 39]
AIDS and Hepatitis	HIV Viral hepatitis B,C	[32, 33, 53]
Abdominal pain	*Helicobacter pylori, Yersinia enterocolitica *	[36]

**Table 2:** The prevalence of selected clinical consequences of SCD.

Clinical event	Prevalence	References
Haemolysis		
Anaemia	Chronic	[57–59]
Cholelithiasis	Prevalence is 40% by adolescence	[60, 61]
Aplastic anaemia	Associated with parvovirus B19 infection	[61–63]
Hyperhemolysis	Limited reports from Africa	[64–67]

Vasoocclusion		
Pain	More than 60% patients Most common cause of admission Frequent pain is a risk factor for mortality	[22, 23, 68, 69]
Acute splenic sequestration (ASS)	Frequently occurs before the age of 3 yrs	[23, 70, 71]
Leg ulcers	Prevalence is 10–25% adults	[72, 73]
Priapism	Prevalence is 10–40% males Occurs frequently in 5–14 years age group	[74]

Organ dysfunction		
Neurological events		
Stroke	Prevalence is 10% in children risk factor for mortality High rate of recurrence Leads to poor quality of life	[75]
Cognitive/silent	Prevalence is 20% Risk factor for overt stroke Leads to impairment of executive function	[76–79]
Retinopathy	Prevalence is >30% in HbSC	[80]
Chest		
Acute chest syndrome (ACS)	Prevalence is 40% Occurs frequently in children Has severe consequences in adults 12.8 per 100-patient years 59	[54–56]
Pulmonary hypertension	Prevalence is 30% Risk factor for mortality	[79, 81–84]
Avascular necrosis of femoral head	Prevalence is 10–50% in adults	[85–87]
Renal disease	Prevalence of chronic renal failure is 5%–20%	[88]

Infections		
Malaria	There is low prevalence of malaria in SCD. However, when malaria occurs in SCD it is associated with increased risk of morbidity due to severe anaemia and mortality	[89, 90]
Bacterial infections	10% children under 5 years	[91]

Modified from [92, 93].

**Table 3:** Summary of study outcomes for hydroxyurea use in adults and children.

Outcome	Impact in adults	Impact in adolescents
Clinical outcomes		
Pain crises	↓↓↓	↓↓
Hospitalisations	↓↓↓	↓↓↓
Blood transfusion therapy	↓↓↓	*↔* (insufficient data)
Acute chest syndrome	↓↓↓	*↔* (insufficient data)
Laboratory markers		
Foetal haemoglobin	↑↑↑	↑↑↑
Haemoglobin	↑↑↑	*↔* (not significantly significant)
Mean corpuscular haemoglobin	↑↑↑	↑↑↑
White blood cell count	↓↓↓	↓↓↓
Prevention of end organ damage		
Brain	*↔*	*↔*
Spleen	*↔*	*↔*
Kidney	*↔*	*↔*
Mortality	↓	*↔*=

↓↓↓: high-grade evidence for decrease; ↓: low-grade evidence for a decrease; ↑↑↑: high-grade evidence for increase; ↑: low-grade evidence for an increase; *↔*: not evaluated/not significantly different/insufficient data. Source [152].
